# Targeting glymphatic-associated pathways for Alzheimer’s disease: a review of non-invasive modulation strategies, mechanisms, and emerging evidence

**DOI:** 10.3389/fimmu.2026.1884893

**Published:** 2026-09-23

**Authors:** Junyu Chen, Wenbo Zhang, Qiang Liu, Zhan Zhang

**Affiliations:** 1 Zhongshan School of Medicine, Sun Yat-sen University, Guangzhou, China; 2 Department of Clinical Medicine, Heze Medical College, Heze, China; 3 Department of Neurology, The First Affiliated Hospital of Guangzhou Medical University, Guangzhou, China; 4 Shandong Provincial Key Laboratory of Brain Science, Institute of Brain Science and Brain-inspired Research, Shandong First Medical University & Shandong Academy of Medical Sciences, Jinan, China; 5 Shandong Institute of Brain Science and Brain-inspired Research, Jinan, China; 6 Department of Geriatric Medicine, Shantou Central Hospital, Shantou, China

**Keywords:** Alzheimer’s disease, aquaporin-4, cerebral waste clearance, glymphatic system, non-invasive modulation

## Abstract

The glymphatic system, a cerebral waste clearance pathway identified over a decade ago, has reshaped understanding of Alzheimer’s disease (AD) pathogenesis by linking impaired brain homeostasis to amyloid-β and tau accumulation. This review synthesizes the biological mechanisms governing glymphatic function, including arterial pulsation, vasomotion, cerebrospinal fluid turnover, norepinephrine oscillations, aquaporin-4 polarization, and meningeal lymphatic drainage, and evaluates emerging non-invasive modulation strategies. Unlike immunotherapies that directly target pathological proteins with risks of amyloid-related imaging abnormalities, non-invasive approaches offer a new perspective, shifting from a target-and-eliminate strategy toward a restore-and-maintain strategy of endogenous clearance. A key insight is that therapeutic efficacy may depend more on engaging multiple mechanistic pathways concurrently than on the specific technology. Notably, preliminary evidence from small exploratory subgroup analyses suggests that APOEϵ4 carriers, despite exhibiting poorer baseline glymphatic function, may show differential responsiveness to certain non-invasive interventions, though these findings are hypothesis-generating and require independent replication in larger, prespecified trials. This review establishes a multidimensional evaluation framework spanning principles, mechanisms, and clinical evidence, providing theoretical support to accelerate translation of these promising strategies into AD therapeutics.

## 1 Introduction

Alzheimer’s disease (AD) is the leading cause of dementia and represents one of the fastest-growing public health crises worldwide. Current projections estimate that the global prevalence of dementia will triple by 2050—an increase that becomes even more pronounced when AD is defined by its underlying biology rather than clinical symptoms alone (1). Neuropathologically, AD is characterized by the progressive accumulation of amyloid beta (Aβ) and hyperphosphorylated tau aggregates in the brain. These pathological species may result from an imbalance between production and clearance, with possibly impaired clearance representing one component of the multifactorial pathogenesis of AD (2–5). Genetic studies have identified over 40 risk loci associated with AD, with the *APOE*ϵ4 allele conferring the strongest susceptibility (1, 6). Moreover, all known dominant mutations causing early-onset AD converge on the amyloidogenic pathway, occurring either in the substrate (amyloid precursor protein, APP) or the protease (presenilin, PSEN1/2) responsible for Aβ generation. PSEN is the catalytic subunit of the γ-secretase. Its mutations result in incomplete cleavage by γ-secretase, leading to an increased proportion of long-chain Aβ such as Aβ42 in the products, which is prone to self-aggregation. Meanwhile, APP mutations make Aβ more prone to forming oligomers, thereby enhancing its toxicity, and they also alter the cleavage efficiency of γ-secretase, working in concert with PSEN mutations to increase the proportion of toxic long-chain Aβ (3, 7).

These insights have fueled the development of immunotherapies targeting Aβ or its precursor protein APP. First-generation anti-amyloid monoclonal antibodies primarily target monomeric Aβ and have failed to demonstrate significant clinical benefits in clinical trials. Currently available second-generation anti-amyloid agents, including aducanumab, lecanemab, donanemab, and gantenerumab, are directed against toxic aggregated forms of Aβ and have been shown to markedly reduce cerebral Aβ deposition (8). Aducanumab received accelerated approval from the U.S. Food and Drug Administration (FDA) in 2021 based on its ability to reduce cerebral Aβ aggregates, while lecanemab has been shown to reduce markers of amyloid in early Alzheimer’s disease and reduce decline on clinical endpoints of cognition and function at 18 months in the phase III Clarity AD trial (NCT03887455) in November 2023. Nevertheless, anti-Aβ antibody therapies are not without limitations. They are associated with an elevated risk of amyloid-related imaging abnormalities (ARIA) (9–12). In addition to neuronal pathology, non-neuronal cells and systemic factors also actively contribute to AD progression. For instance, a distinct population of disease-associated astrocytes (DAAs) emerges at early AD stages (13). These cells, characterized by high GFAP expression and upregulated endfeet markers including AQP4, are integral to the neurovascular unit (NVU) (14). Their reactive gliosis may disrupt AQP4 polarization and interstitial fluid exchange, thereby contributing to the NVU dysfunction that underlies glymphatic impairment. Epigenetic changes, gut–brain axis dysregulation, and chronic sleep disturbances have each been implicated in AD pathogenesis (15–17). Collectively, these observations point to the need for therapeutic strategies that go beyond direct Aβ targeting and address the broader clearance and homeostatic deficits underlying the disease.

The glymphatic or glia-lymphatic system, is a fluid pathway for clearing cerebral wastes (**Figure 1**). Cerebrospinal fluid (CSF) flows from the subarachnoid space along periarterial perivascular spaces (PVS), influxes the brain parenchyma through AQP4-enriched astrocytic endfeet, mixes with brain interstitial fluid (ISF) and circulates within the astrocytic network. It then carries cerebral metabolic wastes and effluxes via the pericapillary-perivenous pathway, ultimately draining into the CSF compartment, venous blood of the dural sinuses, and cervical lymphatic system (18, 19). This cleaning process is critically dependent on the neurovascular unit (NVU) consisting of cerebral microvessels and adjacent neurons and astrocytes. The astrocytic endfeet, membranes surrounding brain capillaries and subpial boundaries, form a key interface mediating neurovascular coupling and glymphatic exchange (20). AQP4 have a preferential enrichment in the endfeet relative to non-perivascular astrocytic membranes, which is called AQP4 polarization. Polarised distribution of AQP4 denotes redistribution away from the endfoot domains rather than reduction in total AQP4 expression (18, 21). The glymphatic system clears neurotoxic metabolites, including Aβ and tau proteins, thereby preserving neural milieu and brain function in AD mice (22). It has also been found that the glymphatic system facilitates the drainage of cerebral Aβ and tau into peripheral plasma among healthy older adults (23). Conversely, glymphatic dysfunction—induced by aging, APOEϵ4 genotype, sleep disruption, or other pathological factors—leads to the accumulation of these toxic byproducts, driving the onset and progression of AD and other neurodegenerative conditions (24–28). One animal study reported that advancing aging triggers a 40% decline in the clearance of intraparenchymally injected Aβ, accompanied by 27% reduction in the vessel wall pulsatility of intracortical arterioles and loss of AQP4 polarization (29). Importantly, mounting evidence suggests that glymphatic impairment occurs at early stages of AD, often preceding significant Aβ deposition, making it not merely a consequence but a potential driver of disease pathology. A recent study revealed that elevated astrocytic Ca^2+^ signaling triggered by Aβ oligomers upregulates astrocyte cholesterol biosynthesis to disrupt AQP-4 polarization, impair glymphatic clearance and drive early cognitive deficits in 5xFAD mice, while suppressing glial calcium activity or inhibiting squalene epoxidase-mediated cholesterol synthesis restores glymphatic function and alleviates amyloid pathology and memory impairment (30). Furthermore, protein aggregates, such as tau and Aβ, can further restrict PVS, exacerbating this dysfunction and generating a vicious cycle of decreased clearance and worsening injury (31). However, though substantial preclinical evidence supports this glymphatic model, the magnitude and relative contribution of glymphatic transport to human AD remain active and further research.

**Figure 1:**
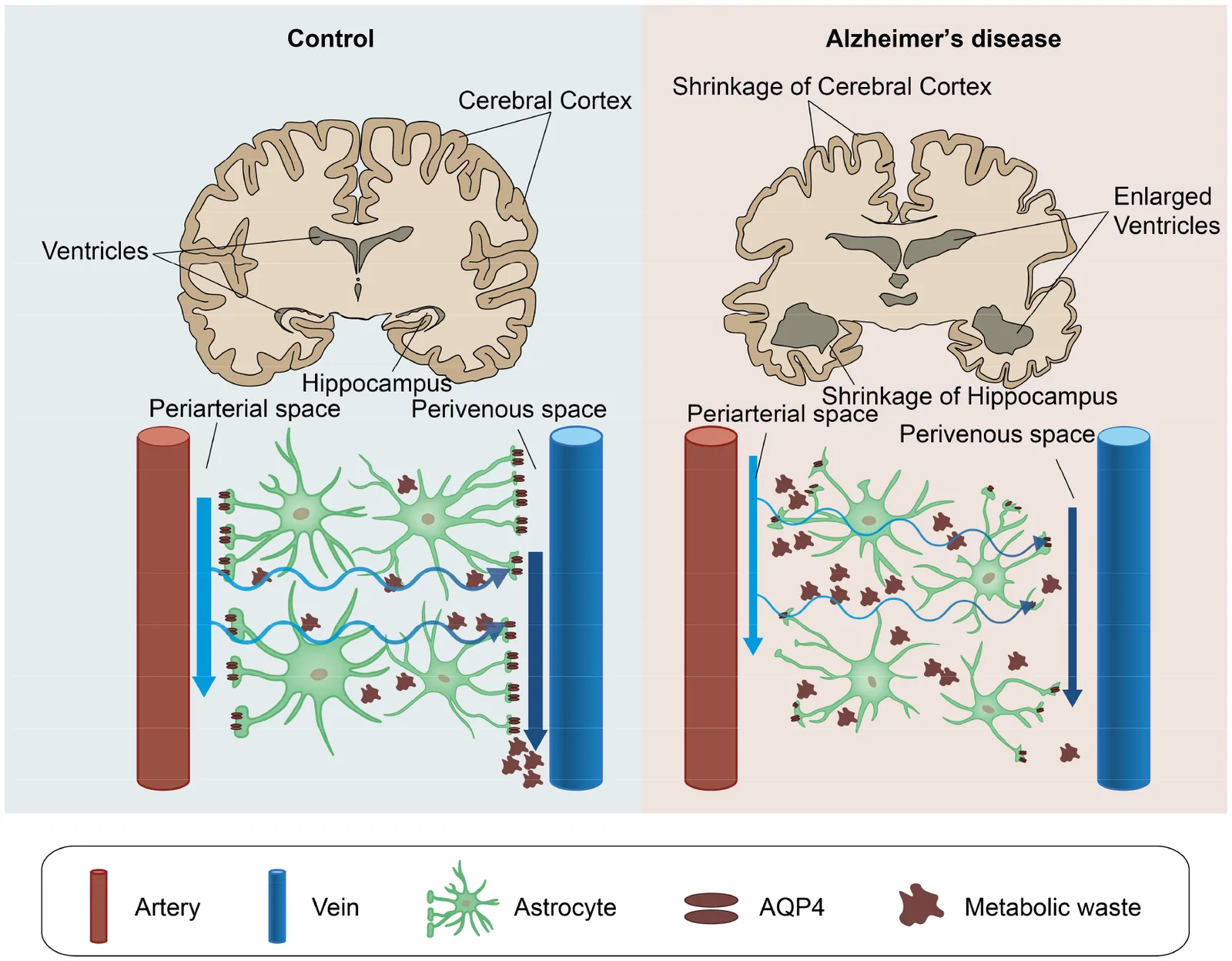
Glymphatic system dysfunction in Alzheimer’s disease. The glymphatic system mediates CSF-ISF exchange and metabolic waste clearance in the healthy brain (left panel). In Alzheimer’s disease (right panel), pathological changes including cerebral and hippocampal atrophy, ventricular enlargement, loss of AQP4 polarization on astrocytic endfeet, and impaired perivascular CSF flow disrupt glymphatic function. These deficits lead to reduced clearance efficiency and accumulation of neurotoxic metabolic waste, such as amyloid-β and tau, which drives disease progression.

Given that glymphatic impairment precedes and exacerbates AD pathology, restoring glymphatic function represents a fundamentally different and highly promising therapeutic avenue. However, directly targeting the glymphatic system poses unique challenges, as its activity is governed by a complex interplay of multiple intrinsic drivers—including arterial pulsation, vasomotion, CSF turnover, norepinephrine oscillations, and AQP4 polarization—along with extrinsic modulators such as intracranial pressure and meningeal lymphatic drainage. The fluid in the subarachnoid space can follow multiple clearance routes: it can be recirculated through the glymphatic pathway, empty into the venous blood of the dural sinuses, or drain into the meningeal and perineural lymphatic systems (19). This complexity calls for interventions that can holistically modulate these multiple determinants rather than targeting a single molecular pathway. Non-invasive modulation of the glymphatic system offers a particularly attractive solution. Unlike invasive or pharmacological strategies that often carry risks such as ARIA, require repeated hospital visits, or address only isolated aspects of pathology, non-invasive techniques may safely, repeatedly, and conveniently engage multiple mechanistic routes to enhance glymphatic clearance and alleviate AD pathology. These approaches may contribute to the refinement of the current AD treatment paradigm from symptom modification or single-target intervention toward the restoration of endogenous waste clearance capacity—a strategy that could be applicable across different stages and even genetic backgrounds of AD.

This review therefore aims to establish a multidimensional evaluation framework for non-invasive glymphatic modulation—spanning technical principles, underlying mechanisms, and clinical efficacy. By synthesizing current evidence and identifying key knowledge gaps, we seek to provide theoretical support and practical guidance to accelerate the translation of these promising strategies from bench to bedside.

## 2 Modulatory mechanisms of the glymphatic system: biological basis for non-invasive interventions

Given the pivotal role of the glymphatic system in AD pathogenesis, elucidating its modulatory mechanisms is essential for understanding how non-invasive therapies exert their effects. To elaborate on its regulatory mechanism more concisely, we constructed a conceptual model categorizing these factors into intrinsic driving factors and extrinsic regulatory factors (**Figure 2**). The functional status of the glymphatic system—under both physiological and pathological conditions—is jointly determined by intrinsic driving factors that originate within the NVU and extrinsic modulatory factors that arise from the surrounding physical and anatomical environment. This conceptual framework not only clarifies the biology of cerebral waste clearance but also reveals distinct nodes amenable to non-invasive intervention.

**Figure 2:**
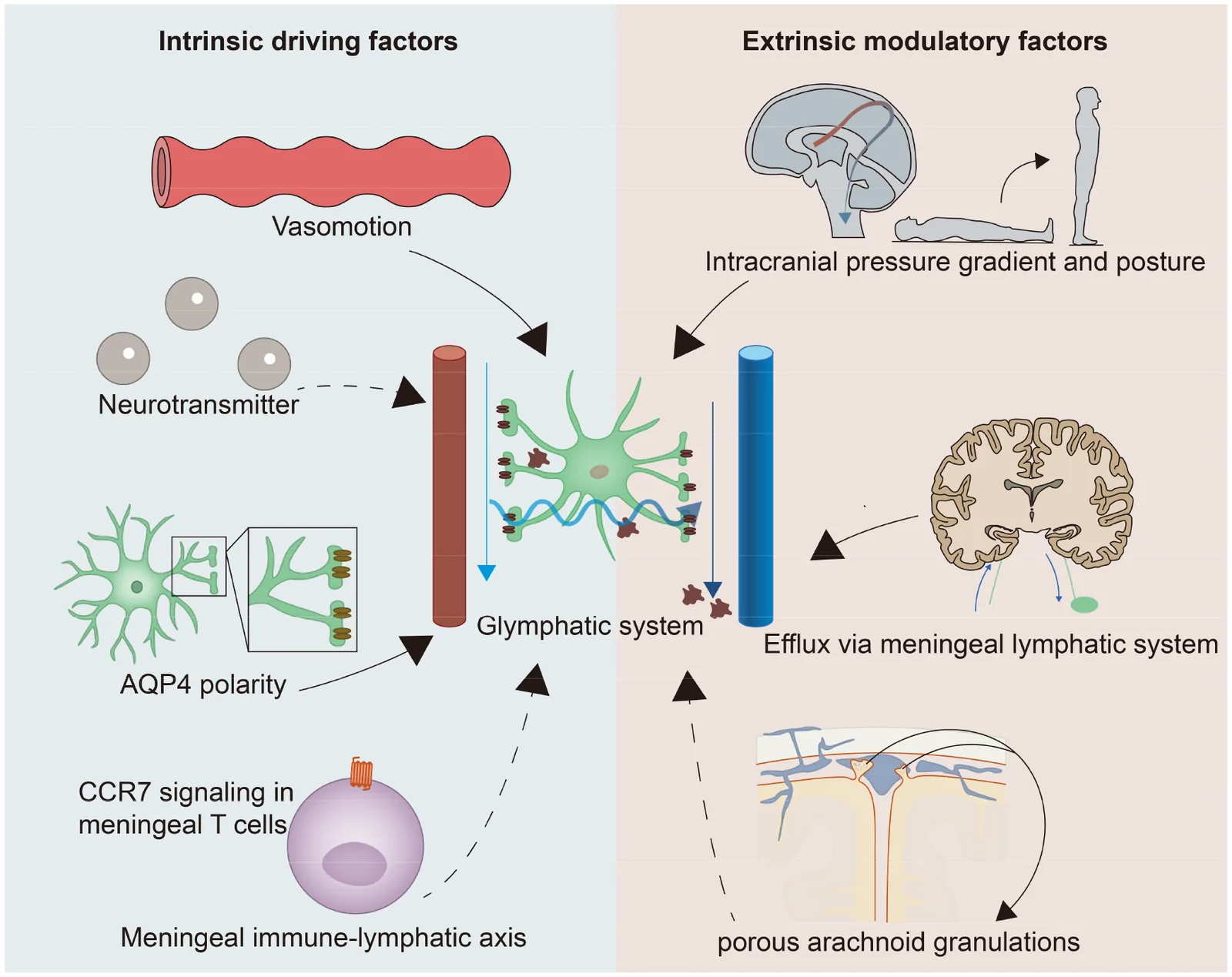
Mechanistic regulators of glymphatic function. Glymphatic clearance is governed by a coordinated interplay of intrinsic driving factors and extrinsic modulatory factors. Intrinsic drivers include vascular vasomotion, neurotransmitter signaling, AQP4 distribution on astrocytic endfeet, and the meningeal immune-lymphatic axis (via CCR7 signaling in meningeal T cells). Extrinsic modulators encompass intracranial pressure gradients and body posture, efflux through the meningeal lymphatic system, and fluid dynamics regulated by porous arachnoid granulations. Together, these factors shape CSF-ISF exchange and metabolic waste clearance within the glymphatic network.

### 2.1 Intrinsic driving factors

Intrinsic drivers refer to endogenous biomechanical and molecular signals that directly power and regulate glymphatic flow. Among these, the dominant hypothesis holds that arterial pulsation serves as the primary physical force driving CSF flow within the PVS (18). Experimental evidence from mouse models demonstrates that internal carotid artery ligation reduces arterial pulsation by 50% and proportionally impairs glymphatic inflow (32, 33). Conversely, pharmacologically enhanced arterial pulsation via the adrenergic agonist dobutamine significantly augments CSF influx in mice (32). Bilateral common carotid artery stenosis similarly diminishes cortical pulsation and selectively disrupts glymphatic drainage in vulnerable regions such as the dorsolateral cortex and hippocampal CA1 area (34). Impaired CSF flow has also been verified to be correlated with defective cerebral clearance in healthy adult humans. It also demonstrates that cardiac cycle was the main driving force of CSF mobility rather than respiration, and shows that CSF mobility in the visual cortex could be enhanced through entraining vasomotion at 0.1 Hz (35). These findings establish vascular hemodynamics as a proximal mechanical determinant of glymphatic function—and a potential target for hemodynamic-based non-invasive therapies.

Beyond passive mechanical forces, neurotransmitter-mediated neural signaling actively regulates glymphatic clearance by modulating neurovascular dynamics. In particular, slow oscillations of norepinephrine during non-rapid eye movement sleep have been identified as a core driver of glymphatic clearance. In mice, these norepinephrine oscillations are highly synchronized with cerebral blood volume and CSF flow, whereas zolpidem, a sleep aid that suppresses norepinephrine oscillations, directly reduces glymphatic flow in rodent models (27). In AD model mice, sleep deprivation fails to elicit the normal enhancement of norepinephrine oscillations, and this blunted response correlates directly with brain Aβ accumulation, suggesting that loss of noradrenergic rhythmicity increases glymphatic vulnerability to sleep disruption in early AD (36).

The modulatory mechanism of the glymphatic system is also associated with the turnover rate of CSF. CSF is primarily secreted by the choroid plexus and circulates within the subarachnoid space and PVS via the aforementioned fluid pathways. Based on the model established by Hornkjøl describing the relationship between tracer diffusion in CSF and the human brain interstitium, a review concluded that CSF diffusion and clearance are delayed when CSF production declines or cerebral atrophy induces a pathological expansion in CSF volume (37, 38). In patients with AD presenting cognitive symptoms, larger choroid plexus volume correlates with greater severity of cognitive impairment across the AD spectrum. This finding validates the aforementioned hypothesis, suggesting that impaired CSF turnover deteriorates glymphatic function and exacerbates AD pathology (39).

At the molecular level, AQP4 constitutes the core transmembrane conduit for fluid transport and functional maintenance of the glymphatic system. The polarized distribution of AQP4 on astrocytic endfeet follows a circadian rhythm, peaking during the rest phase in mice (corresponding to sleep in humans), thereby underpinning the circadian rhythm of glymphatic clearance. AQP4 knockout completely abolishes circadian differences in both glymphatic influx and efflux (40). Sleep fragmentation reduces AQP4 expression, while the α2-adrenergic agonist dexmedetomidine upregulates AQP4 and rescues glymphatic dysfunction and related behavioral deficits induced by sleep deprivation (26, 41). Moreover, a cross-sectional study in cognitively normal older adults revealed that genetic variants of AQP4 modulate the relationship between sleep quality and brain Aβ burden, hinting that AQP4 serves not only as an effector molecule but also as a genetic susceptibility factor for glymphatic dysfunction (42).

Finally, aging-related immune dysregulation represents an emerging intrinsic factor. Aging induces a significant decline in CCR7 expression in meningeal T cells, which impairs immune cell efflux through lymphatic vessels and leads to pathological accumulation of meningeal effector and regulatory T cells. In 5xFAD AD model mice, CCR7 knockout exacerbates neurovascular damage, microglial activation, and Aβ deposition, while anti-CD25 antibody treatment alleviates meningeal regulatory T cell abnormalities and improves cognitive function in aged mice (25). This immune–lymphatic axis highlights how intrinsic immunological aging can secondarily compromise glymphatic clearance.

### 2.2 Extrinsic modulatory factors

Extrinsic modulators encompass physical forces and anatomical pathways that originate outside the neurovascular unit but profoundly influence glymphatic function. The intracranial pressure gradient is a primary extrinsic physical force. A small longitudinal pressure gradient simultaneously accounts for both oscillatory and mean flow around cerebral arteries, acting as the core driver of perivascular mean flow that propels CSF from PVS into brain parenchyma and onward to meningeal lymphatic vessels (43). Body posture dynamically modulates this gradient: in the supine position, CSF flow in the optic nerve subarachnoid space is directed outward through the optic nerve sheath, whereas in the upright position, flow redirects toward the intracranial subarachnoid space (44). This posture-dependent modulation has direct translational implications for non-invasive interventions, as head positioning during sleep or therapy could be optimized to enhance glymphatic clearance.

The meningeal lymphatic system constitutes another critical extrinsic modulator. In both mice and humans, meningeal lymphatic vessels are located along the dural venous sinuses dorsally and along cranial nerves ventrally, establishing direct anatomical connections with the deep cervical lymph nodes (dCLNs) via skull base foramina. These pathways enable extracranial clearance of metabolic waste (45, 46). Functional impairment of meningeal lymphatics—whether genetic or surgical—markedly reduces macromolecule clearance. Transgenic mice with hypoplastic meningeal lymphatics exhibit complete disruption of CSF transport to dCLNs and profound retention of brain macromolecules (46, 47). In AD model mice, ligation of dCLNs (which blocks the extracranial drainage pathway) aggravates Aβ deposition, impairs AQP4 polarity, exacerbates neuroinflammation, and accelerates cognitive decline (48). Conversely, therapeutic dilation of meningeal lymphatics has emerged as a promising non-invasive strategy.

Importantly, arachnoid granulations may serve as a key coupling node between the glymphatic system and meningeal lymphatic vessels. Their porous microstructure forms a trans-arachnoid pathway for CSF, suggesting uncharacterized functions in mechanical CSF filtration (49). However, recent evidence suggests that arachnoid granulations may be more involved in immune surveillance at the brain surface than serving as a major site for bulk CSF drainage, while the predominant outflow path for CSF appears to be the meningeal lymphatic system (49, 50).

## 3 Non-invasive modulation strategies: from technology to evidence

Building upon the mechanistic framework of intrinsic and extrinsic modulators, this section evaluates seven non-invasive intervention strategies that target the glymphatic system (**Figure 3**). For each modality, we examine its core technological principle, the specific pathways through which it modulates glymphatic function, and the evidence from preclinical and clinical studies. A summary of the relevant evidence is provided in **Table 1**.

**Figure 3:**
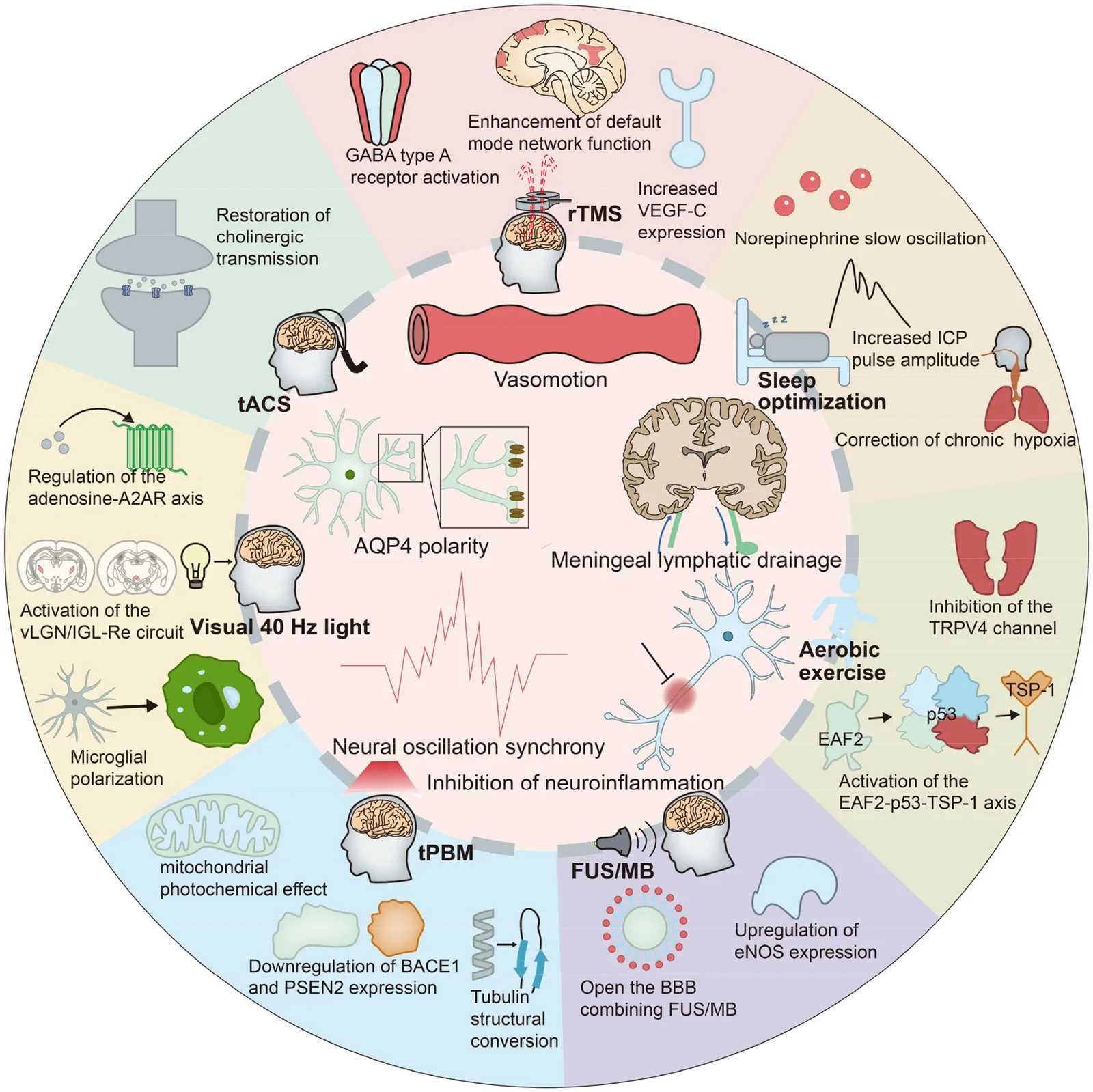
Mechanistic overview of non-invasive interventions targeting the glymphatic system. This schematic summarizes the core pathways through which seven non-invasive interventions enhance glymphatic function, targeting key regulators including vasomotion, AQP4 polarity, meningeal lymphatic drainage, neural oscillation synchrony, and neuroinflammation. rTMS modulates GABAergic signaling and meningeal lymphatic flow; tACS restores cholinergic transmission; 40-Hz light stimulation regulates the adenosine-A2AR axis and promotes microglial phagocytosis; tPBM improves mitochondrial function and reduces AD-related gene expression; LIFU enhances vascular function and enables transient BBB opening; aerobic exercise modulates the TRPV4 and EAF2-p53-TSP-1 pathways; and sleep optimization restores norepinephrine oscillations and intracranial pressure dynamics.

**Table 1:** Evidence of non-invasive modulation strategies.

First Author(Ref.)	Intervention Modality	Study Design	Population/Model	Sample Size (Interv./Cont.)	Intervention Parameters	Comparator	Glymphatic Assessment Methods	Direct or Indirect	Main Glymphatic Outcomes	Other Outcomes	Safety	Glymphatic Evidence*
Cheng Wu (52)	cTBS	Sham-controlled preclinical animal study	Male APP/PS1 mice, 7–8 months old	6/6	50-Hz bursts repeated at 5 Hz; continuous 40-s train; 0.75 T; once daily for 4 weeks; whole-brain stimulation	Sham CTBS	Intracisternal FITC-dextran tracer with *in vivo* two-photon	Direct	CTBS increased CSF tracer influx into the perivascular space and interstitial tissue, indicating enhanced CSF–ISF exchange	Increased AQP4 perivascular polarization; reduced cortical and hippocampal Aβ1-40/Aβ1–42​ deposition; improved Morris water-maze spatial memory	No adverse events reported	Very high
Cheng-Chang Yang (53)	iTBS	Double-blind, randomized, sham-controlled parallel-group clinical trial	Participants with amnestic MCI or very mild AD; CDR = 0.5	30/30	50-Hz bursts repeated at 5 Hz; 600 pulses/run; 3 runs/session; 1,800 pulses/session; 20 min/session; 80% resting motor threshold; left DLPFC; 10 sessions over 2 weeks	Sham iTBS; sham group later received active iTBS	DTI-ALPS index	Indirect	No significant immediate or delayed change in the ALPS index after iTBS	MMSE improved at week 6 after active iTBS, but no immediate cognitive benefit was observed	No adverse events reported	High
Xin Zhang (54)	iTBS	Prospective randomized, double-blind, sham-controlled parallel-group clinical trial	Patients aged 50–80 years with biomarker-supported AD	10/10	50-Hz bursts repeated at 5 Hz; 600 pulses per block; four bilateral blocks with 5-min intervals; 1,200 stimuli/hemisphere/session; 20 weekday sessions over 4 weeks; 48,000 stimuli in total	Sham iTBS	DTI-ALPS index	Indirect	Active iTBS increased the DTI-ALPS index compared with sham	Improved CDR-SB, ADAS-Cog, and MMSE; decreased EEG θ power and increased beta power; improved DMN functional organization	Mild same-day headache in 2/10 active participants; no adverse events in the sham group; no serious adverse events reported	High
Mark H. Sundman (59)	iTBS/cTBS	Randomized-order, sham-controlled, within-subject repeated-measures study	Older adults with MCI; 13 APOE ϵ4 carriers and 23 non-carriers	n=36	Left parietal cortex stimulation; 600 pulses/session; iTBS delivered as 50-Hz bursts at 5 Hz with 8-s intertrain intervals; cTBS delivered continuously; 10 weekday sessions per condition; approximately 1-month washout	Sham stimulation	DTI-ALPS index	Indirect	TBS increased the sham-controlled DTI-ALPS index, particularly in APOE ϵ4 carriers, who had lower baseline values	Increased ALPS values correlated with improved face-name associative memory in APOE ϵ4 carriers	style="background-color:#ddd9c3"No adverse events reported	High
Alberto Benussi (64)	tACS	Randomized, double-blind, sham-controlled crossover trial	Patients with biomarker-supported AD	60/60 crossover participants	40 Hz; 1.5 mA peak-to-baseline, 3.0 mA peak-to-peak; 0.09 mA/cm²; precuneus stimulation; one 60-min session	Identical electrode montage; current ramped down after 60s	No glymphatic-specific assessment	N/A	No direct glymphatic outcome	Improved RAVLT immediate and delayed recall and face-name associative memory; increased gamma activity; improved short-latency afferent inhibition, indicating enhanced cholinergic transmission; response was influenced by APOE genotype and disease severity	No adverse events reported	Very low
Giulia Sprugnoli (66)	tACS	Two open-label pilot studies	Patients with mild-to-moderate AD; mean MMSE 23.5	15/0	40 Hz; up to 2 mA per electrode and 4 mA total; 1 h/day on weekdays; 2-week unilateral temporo-frontal or bitemporal stimulation, or 4-week bitemporal stimulation	No comparator	Cerebral blood flow	Indirect	Increased cerebral blood flow mainly in the temporal lobes; the 4-week bitemporal protocol increased perfusion in bilateral temporal, entorhinal, and hippocampal regions	Temporal perfusion changes correlated with delayed episodic-memory performance and gamma-power changes	Mild tingling (10/15), scalp irritation (7/15), visual sensations (8/15), and headache (5/15); no epileptiform activity or serious adverse events	Moderate
Tao Wang (67)	tACS	Prospective randomized, double-blind, sham-controlled clinical trial	Patients aged 45–75 years with mild AD	23/23	40 Hz; 15-mA current intensity; 1 h/session; 30 sessions over 3 weeks; sessions delivered twice daily on weekdays	Sham tACS	Resting-state functional MRI with seed-based hippocampal and cortical functional-connectivity analysis	N/A	No direct glymphatic outcome; active tACS increased functional connectivity between the left hippocampus and left middle cingulate and middle frontal gyri	MMSE and MoCA improved at week 3; increased posterior/anterior DMN and visual-network connectivity; some network effects persisted at month 3	No adverse events reported	Very low
Yi Tang (68)	tACS	Single-center, double-blind, randomized, sham-controlled clinical trial	Patients aged 45–75 years with mild AD	23/23	40 Hz; 15-mA peak-to-peak amplitude; 1 h/session; 30 sessions over 15 consecutive days; two sessions/day with ≥4-h intervals	Sham tACS	Simultaneous EEG-fMRI assessing hippocampal theta–gamma coupling and hippocampal fALFF	N/A	No direct glymphatic outcome	MMSE and MoCA improved immediately but not at 3 months; delayed recall improved; hippocampal coupling correlated with MMSE and delayed recall	Well tolerated; one participant reported transient tingling in the active group and one reported transient itching in the sham group; no serious adverse events reported	Very low
Mitchell H. Murdock (71)	visual and auditory stimulation	Controlled preclinical animal study	6-month-old 5XFAD mice; additional VIP-Cre/5XFAD and AQP4-manipulated mice	Representative CSF-tracer assay: 4/4; amyloid assay: 8/10	40-Hz audiovisual stimulation; LED: 60 W; 12.5-ms on/12.5-ms off; auditory tone: 10 kHz, 60 dB; 1 h acute exposure; some behavioral experiments used 1 h/day repeatedly	No stimulation, 8-Hz stimulation, or 80-Hz stimulation	Cisterna magna fluorescent CSF-tracer infusion with ex vivo confocal imaging and awake *in vivo* two-photon imaging of CSF influx; two-photon assessment of ISF efflux	Direct	40-Hz stimulation increased cortical CSF influx and ISF efflux, arterial pulsatility, AQP4 polarization, and meningeal lymphatic-vessel diameter; effects were attenuated by AQP4 or VIP-neuron inhibition	Reduced cortical amyloid burden and increased amyloid accumulation in deep cervical lymph nodes; improved novel-object recognition in chronic-treatment experiments	No adverse events reported	Very high
Wen Wu (76)	40-Hz blue-light flicker	Controlled preclinical animal study with pathway and AQP4-blockade experiments	4- and 14-month-old 5XFAD mice and WT mice	Representative 4-month cohort: 10/10	Blue LED, 472 nm; 40 Hz; 0.03 mW/cm²; 160–180 lux; 1 h/day for 14 consecutive days	Sham lymphatic ablation, vehicle treatment, and chemogenetic pathway controls	Intracisternal tracer imaging in brain parenchyma and deep cervical lymph nodes; representative MRI assessment used intracisternal Gd-DOTA with dynamic T1-weighted MRI	Direct	Blue light increased CSF-tracer perfusion and clearance to deep cervical lymph nodes and increased the hippocampal Gd-DOTA influx rate; AQP4 inhibition abolished the effect	Improved Y-maze working memory, novel-object recognition, and motivational behaviors; restored perivascular AQP4 polarization; reduced soluble hippocampal Aβ42 and abnormal lipid accumulation	No adverse events reported	Very high
Annabelle C. Singer (125)	Noninvasive 40-Hz white-light flicker	Methodological protocol based on previous preclinical experiments; not an independent efficacy trial	5XFAD and APP/PS1 mice	protocol paper with variable sample sizes	White LED light, 4,000 K; 100–480 lux; 40 Hz; 50% duty cycle; 12.5-ms light on/12.5-ms off; usually 1 h exposure followed by 1-h holding; may be repeated daily for 1–7 days	Dark, continuous light, randomized stimulation, or non-gamma frequencies such as 20 or 80 Hz	No glymphatic-specific assessment described; the protocol focuses on microglial morphology, Aβ immunohistochemistry, CLARITY, and Aβ–microglia colocalization	N/A	No direct glymphatic outcome; the protocol is designed to assess microglial engulfment and Aβ reduction	Expected increased microglial engulfment and Aβ–microglia colocalization; reduced soluble Aβ and, after repeated exposure, reduced plaque number and size	No adverse events reported	Low
Sang-Seo Park (80)	Combined aerobic exercise combined 40-Hz light flicker; single-modality exercise and flicker arms	Controlled preclinical, multi-group intervention study	Five-month-old male 3×Tg-AD mice and WT mice	10/10 for the combined-treatment versus AD-model comparison; five groups in total	Treadmill exercise once daily, 6 days/week for 12 weeks; intensity progressed from 10 to 13 m/min; 40-Hz light flicker was delivered for the same duration as exercise	AD-only, exercise-only, 40-Hz flicker-only, and nontransgenic control groups	No glymphatic-specific assessment; hippocampal Aβ, tau, GFAP, Iba1, cytokines, mitochondrial function, apoptosis, and synaptic proteins were measured	N/A	No direct glymphatic outcome;	Combined exercise and flicker produced the greatest improvement in Morris water-maze spatial memory and step-through long-term memory	No adverse events reported	Low
Mihály Hajós (127)	Gamma stimulation using the Spectris device	Exploratory biomarker analysis nested within the FLICKER clinical trial; delayed-start comparison	Amyloid-positive individuals with MCI due to AD	Not reported	Daily 1-h Spectris treatment for 8 weeks; detailed device intensity NR	Delayed-start/no-stimulation condition	Unbiased CSF proteomic profiling	N/A	No direct glymphatic outcome; 110 of 2,951 CSF proteins showed nominally significant treatment-related changes	Altered CSF proteins were mainly related to myelination, synaptic/ne neuronal function, complement/neuroimmune pathways, and lipid transport; 60 proteins increased and 50 decreased	No adverse events reported	Low
Ariel Motsenyat (84)	tPBM/iPBM	Within-subject repeated-measures, dose-response physiological study	Healthy young adults aged 20–32 years	45/0	808- or 1,064-nm light; 10 or 40 Hz; tPBM: 100–200 mW/cm²; iPBM: 5–9 mW/cm²; 50% duty cycle; 4 min per stimulation block; multiple protocols	No comparator	Dual-echo pseudo-continuous arterial-spin-labeling/BOLD fMRI of CSF-related regions	Indirect	PBM produced an immediate increase in intracranial CSF volume and a reduction in CSF inflow, consistent with enhanced CSF displacement/outflow; responses were dose-dependent and comparable between iPBM and tPBM despite lower iPBM irradiance	Lighter skin pigmentation was associated with a stronger tPBM CSF response	No adverse events reported	High
Horner (126)	tPBM/iPBM	Single-patient uncontrolled case report	A 59-year-old man with insulin-dependent T2DM, heterozygous APOE ϵ4, and MCI/mild AD	n=1	810-nm near-infrared PBM; 40 Hz; 50% duty cycle; transcranial and intranasal delivery; 20 min/session, 3 times/week for 10 weeks; combined with a clinically prescribed ketogenic diet and moderate exercise	No comparator	No glymphatic-specific assessment; cognitive, metabolic, and peripheral biomarker assessments were performed	N/A	No direct glymphatic outcome	MoCA improved from 20/30 to 26/30; HOMA-IR decreased by 83%; HbA1c decreased from 9.4% to 6.4%; triglyceride/HDL ratio and other metabolic markers improved	No adverse events reported	Very low
Kumiko Eguchi (99)	low-intensity pulsed ultrasound (LIPUS)	Controlled preclinical study	5XFAD mice and BCAS-induced vascular-dementia mice	AD model: approximately 11–15/11–15;	1.875 MHz carrier frequency; 6-kHz pulse-repetition frequency; 32 cycles; ISPTA= 90 mW/cm²; 20 min/session for 3 sessions; effects followed for up to 3 months	Disease-model and sham operation	Laser-speckle imaging of cerebral blood flow CBF	Indirect	LIPUS improved or preserved CBF in dementia-model mice, with the effect lasting up to 84 days in 5XFAD mice; eNOS activation was required for the beneficial vascular response	Improved Y-maze performance; reduced cortical Aβ plaque burden and Triton-soluble Aβ42; reduced microgliosis; increased eNOS and neurotrophin expression	No adverse events reported	Moderate
Hyeonseok Jeong (129)	LIFU	Prospective uncontrolled pilot clinical study	Patients with early-stage, amyloid-positive AD; mean age 72.4 years; CDR 0.5–1	10/0	250-kHz ultrasound; 1-ms tone bursts; 500-Hz pulse-repetition frequency; 50% duty cycle; ISPPAISPPA​ = 3.0 W/cm² and ISPTAISPTA​ = 1.5 W/cm²; 20 min/session; six sessions over 2–3 weeks; left DLPFC target	No comparator	No glymphatic-specific assessment; resting-state fMRI was used for functional-connectivity analysis	N/A	No direct glymphatic outcome	Memory-domain performance improved; increased DLPFC–perirhinal connectivity correlated with memory improvement; global MMSE-2 did not significantly improve	No adverse events reported	Very low
H Xiaoxia Li (128)	BCZT piezoelectric nanoparticle-mediated 40-Hz transcranial focused ultrasound	Controlled preclinical animal study with electrophysiological, imaging, and mechanistic experiments	Male APP/PS1 mice aged 2–2.5 months and age-matched WT mice	Representative LFP analysis: 4/4; sample size varied by assay	842-kHz ultrasound; 3-ms pulses; 40-Hz repetition frequency; 60 min/day for 7 consecutive days; ISPTAISPTA​ = 720 mW/cm² and ISPPAISPPA​ = 190 W/cm²; 1 μL BCZT nanoparticles injected into hippocampal CA3	Sham stimulation, saline plus ultrasound, or no stimulation, NF-κB inhibitor.	No glymphatic-specific assessment; hippocampal CA3 local-field-potential recording was the representative neural-function method	N/A	No direct glymphatic outcome; BCZT-mediated ultrasound restored CA3 gamma power and theta–gamma phase-amplitude coupling	Restored AMPAR-mediated synaptic transmission and AMPAR/NMDAR ratio; increased GluR1 through NF-κB activation; enhanced hippocampal–medial prefrontal functional connectivity; reduced Aβ oligomer burden	No adverse events reported	Very low
Ali R. Rezai (130)	FUS.MB	Open-label, prospective phase II clinical trial	Patients with early AD; 5 females and 1 male, aged 55–73 years	6/0	220-kHz FUS; hippocampus/entorhinal cortex; up to five 5×5×7-mm targets; three sessions at 2-week intervals; 17 treatment procedures in total	No comparator	Contrast-enhanced T1-weighted MRI with gadobutrol to quantify BBB opening and closure	N/A	No direct glymphatic outcome; BBB opening occurred in 95% ± 4% of the targeted volume and closed within 24 h	No meaningful cognitive or neurological worsening	No adverse events reported	Very low
Ali R. Rezai (101)	FUS/MB	Open-label, prospective clinical trial with 6–12 months of follow-up	Patients with mild AD, aged 55–76 years	10/33	220-kHz FUS; hippocampus, entorhinal cortex, frontal lobe, and parietal lobe; three sessions separated by 2 weeks	No sham intervention; age- and sex-matched ADNI patients served as an external observational cognitive comparator	Contrast-enhanced MRI to quantify BBB opening and closure	N/A	No direct glymphatic outcome; BBB opening involved an average of 82% of the targeted region and closed within 24–48 h	Focal β-amyloid PET burden decreased by approximately 14% on the Centiloid scale; cognitive decline was comparable to ADNI controls	One transient hippocampal edema resolved within 72 h	Low
Xiao-fei He (105)	Voluntary wheel running	Controlled preclinical animal study	Male aged C57BL/6J and Thy1-GFP mice, 14–16 months old	12/12 C57BL/6J mice; 6/6 Thy1-GFP mice	Voluntary wheel running using a 16-cm wheel; free access for 6 weeks	Standard cages without running wheels	Intracisternal or intraparenchymal FITC-dextran tracing with *in vivo* two-photon imaging of CSF–ISF exchange and ISF drainage	Direct	Exercise accelerated paravascular CSF–ISF exchange and ISF drainage, increased tracer delivery to deep cervical lymph nodes, and improved AQP4 polarization	Improved Morris water-maze performance; reduced Aβ1–40/Aβ1–42, GFAP-positive astrocytes, and IBA1-positive microglia; increased dendritic spines and PSD95	No adverse events reported	Moderate
Yan Chen (107)	Treadmill aerobic exercise	Controlled preclinical animal study with mechanistic genetic and *in vitro* experiments	5×FAD mice and age-matched wild-type mice; approximately 5.5–6.5 months old	Representative experiments: 6/6; sample size varied by assay	Uphill treadmill at 10°circ; twice daily; 90 min/session; progressive speed beginning at 8 m/min and increasing by 1 m/min every 20 min; 30 days	Identical housing conditions without treadmill exercises	Intracisternal tracer and intrahippocampal fluorescent Aβ1–42, assessed by *in vivo* two-photon imaging of LYVE-1-positive meningeal lymphatics and deep cervical lymph-node drainage	Direct	Exercise increased meningeal lymphatic vessel coverage, diameter, sprouts, zipper-like junctions, and Aβ drainage to deep cervical lymph nodes; perivascular AQP4 localization was also improved	Improved short-term learning and memory; reduced Aβ plaques, reactive gliosis, astrocyte senescence, and CSF/hippocampal TSP-1; effects were associated with downregulation of the EAF2–p53–TSP-1 pathway	No adverse events reported	Very high
Yun Liu (108)	Voluntary wheel running	Controlled preclinical study with genotype- and age-dependent comparisons	Male WT, APP/PS1, and AQP4-deficient APP/PS1 mice; intervention initiated at 3 or 7 months of age	12–13/12–13 per group; tracer subgroup: 6/6 per group	Voluntary running wheel, 4 h/day, 5 days/week for 8 weeks; 12.5-cm wheel	Locked wheels	Intracisternal tracer with ex vivo fluorescence quantification of parenchymal and perivascular tracer penetration	Direct	Exercise increased glymphatic tracer penetration and perivascular transport in 5-month-old APP/PS1 mice, but not in older APP/PS1 mice or AQP4-deficient mice	Early exercise reduced Aβ plaque burden, reactive gliosis, and neuroinflammation and improved Morris water-maze, Y-maze, and object-recognition performance; benefits were absent with AQP4 deficiency or advanced pathology	No adverse events reported	Very high
Mingyue Li (109)	Voluntary wheel exercise	Controlled preclinical study with pharmacological mechanistic validation	Female C57BL/6J mice with DSS-induced chronic colitis, 18 months old	Behavioral assessments: 11/11 per group; glymphatic imaging: 3/3 per group	Freely accessible 16-cm running wheel for 4 weeks; TRPV4 agonist GSK1016790A, 10 μg/kg/day intraperitoneally, was used in the mechanistic group	Water-treated controls, DSS-only mice, and exercise plus DSS plus vehicle controls; exercise plus DSS plus GSK1016790A	Intracisternal FITC-dextran with *in vivo* two-photon imaging and ex vivo whole-brain fluorescent-tracer quantification	Direct	Exercise accelerated FITC tracer clearance and increased glymphatic transport; the effect was abolished by TRPV4 activation and accompanied by increased AQP4 polarization	Improved Morris water-maze, novel-object recognition, and open-field behavior; reduced brain Aβ*1–40/Aβ*1–42, neuronal apoptosis, microglial activation, and pro-inflammatory cytokines; inhibited TRPV4-related astrocytic calcium hyperactivity	No adverse events reported	Very high
Lauren M. Hablitz (110)	Anesthetic modulation	Controlled comparative preclinical animal study with physiological correlation analyses	C57BL/6 mice, 3–5 months old	CSF-tracer cohorts: ketamine/xylazine (n=36), isoflurane/dexmedetomidine (n=14), pentobarbital (n=27), α-chloralose (n=20), Avertin (n=27), isoflurane (n=23)	Ketamine/xylazine: 100/20 mg/kg intraperitoneally; pentobarbital: 60 mg/kg; Avertin: 120 mg/kg; α-chloralose: 80 mg/kg; isoflurane: 1–2%; isoflurane/dexmedetomidine: isoflurane plus dexmedetomidine 0.015 mg/kg; CSF tracer circulated for 30 min	Each anesthetic regimen served as a comparator for the other regimens	Intracisternal tracer with ex vivo whole-brain and coronal-slice fluorescence quantification; simultaneous EEG and cardiopulmonary monitoring	Direct	CSF tracer influx was highest under ketamine/xylazine, intermediate under isoflurane/dexmedetomidine and pentobarbital, and lowest under isoflurane, Avertin, and α-chloralose; influx correlated positively with 1–4-Hz delta power and negatively with beta power and heart rate	EEG delta activity and heart rate as physiological correlates of glymphatic influx	No adverse events reported	Very high
Samantha A. Keil (114)	CPAP	Retrospective cross-sectional imaging study with an observational CPAP-treated subgroup	Adults with untreated OSA, CPAP-treated OSA, and healthy controls	14 untreated OSA/12 CPAP-treated OSA/16 healthy controls; PET subgroup (n=25)	CPAP-treated participants had documented CPAP use for at least 6 months before MRI; treatment adherence and exact daily duration were not uniformly available	Healthy controls and untreated OSA participants	Parenchymal CSF fraction mapping, conventional MRI-visible PVS	Indirect	Untreated OSA showed higher ADmask pCSF than healthy controls, indicating altered PVS CSF-like fluid distribution; CPAP-treated participants had pCSF levels comparable to controls; conventional MRI-visible PVS did not differ significantly	Higher ADmask pCSF was independently associated with greater C11-PiB amyloid burden; OSA groups had lower MoCA-Blind scores than controls	No adverse events reported	High
Shiwei Lin (115)	Standard clinical treatment for OSA; uvulopalatopharyngoplasty (UPPP) in the longitudinal subgroup	Prospective cross-sectional comparative study with longitudinal pre–post treatment follow-up	Adults aged 18–60 years with snoring or OSA and healthy controls	105 participants with snoring/OSA: snoring (n=21), mild OSA (n=36), moderate OSA (n=25), severe OSA (n=23); healthy controls (n=50); UPPP follow-up subgroup (n=15)	UPPP for moderate OSA; follow-up MRI and cognitive testing performed 3–6 months after surgery; exact surgical parameters NR	Healthy controls and untreated OSA-severity groups; within-subject comparison	DTI-ALPS, PVS volume fraction and free-water index	Indirect	Severe OSA had higher PVS volume fraction and free-water index and lower DTI-ALPS values than healthy controls; all OSA groups had lower DTI-ALPS than controls; after UPPP, PVS indexes improved, including increased DTI-ALPS	Lower DTI-ALPS correlated with poorer Stroop Color and Word Test performance; after UPPP, processing speed and executive-function measures improved	No adverse events reported	High

Level of Glymphatic Evidence*: Very high: direct tracer-based clearance measurements, High: MRI-derived surrogate measures, Moderate: CSF-BOLD coupling or physiological proxies, Low: molecular or histological correlates, Very low: clinical outcomes without direct measurement of glymphatic function. Color corresponds to different intervention methods.

### 3.1 Repetitive transcranial magnetic stimulation

Repetitive transcranial magnetic stimulation (rTMS) and its derivative theta burst stimulation (TBS)—including continuous TBS (cTBS) and intermittent TBS (iTBS)—have emerged as potential therapeutic approaches for AD and prodromal mild cognitive impairment (MCI). Their core mechanism involves inducing action potentials in cortical or cerebellar neurons through alternating magnetic fields, thereby modulating brain network activity, molecular pathway expression, and cerebrovascular-CSF dynamics (51–54).

rTMS/TBS regulate the glymphatic system through convergent multi-pathway effects. High-frequency rTMS suppresses pathological glial activation and neuroinflammation in the 5×FAD mouse brain, which may help establish a stable microenvironment for glymphatic function while reducing neuronal loss and restoring neuronal activity, thereby indirectly enhancing network-level regulation of glymphatic clearance (51). In APP/PS1 mice, cTBS upregulates AQP4 expression and restores its polarized distribution, enhances perivascular CSF-ISF exchange efficiency, and improves glymphatic drainage rate, leading to reduced Aβ deposition (52). cTBS promotes glymphatic clearance via GABA type A receptor activation in an AQP4-dependent manner—an effect abolished by either AQP4 knockout or GABA-A receptor blockade, suggesting GABA as a key neurotransmitter mediator of rTMS action on the glymphatic system in preclinical settings (55, 56). Additionally, cTBS specifically upregulates vascular endothelial growth factor-C (VEGF-C) in mouse meningeal tissue, activating VEGFR3 to dilate meningeal lymphatic vessels and enhance glymphatic drainage, and pathway blockade completely abolishes this effect (57, 58). Beyond these central mechanisms, cerebellar iTBS improves whole-brain coupling between blood oxygen level-dependent signals and CSF flow by enhancing default mode network function in AD patients, thereby restoring the physical driving force for glymphatic clearance (54).

In preclinical studies, both high-frequency rTMS and cTBS have demonstrated consistent efficacy in improving glymphatic function, reducing Aβ deposition, and alleviating cognitive impairment in 5xFAD and APP/PS1 mouse models (51, 52). In human studies, TBS has been associated with an increase diffusion tensor imaging analysis along the perivascular space (DTI-ALPS) scores, an indirect imaging surrogate that reflects changes in perivascular water diffusivity rather than a direct measure of glymphatic function. Notably, although APOEϵ4 carriers exhibit poorer baseline glymphatic function, they show significantly greater responses to TBS than non-carriers in a small exploratory cohort study, and the improvement in glymphatic function correlates positively with memory enhancement (59). However, this observation is derived from a subgroup analysis and requires confirmation in larger, adequately powered and prespecified trials. A separate study in MCI and very early AD patients reported that six weeks of iTBS over the left dorsolateral prefrontal cortex produced cognitive improvement with a delayed onset (increased MMSE scores), without significant DTI-ALPS changes during or immediately after the intervention, suggesting that long-term network modulation may operate through distinct mechanisms that are not captured by acute glymphatic measures (53). In a study of patients with typical AD, four weeks of cerebellar iTBS significantly altered BOLD-CSF coupling and reduced Clinical Dementia Rating Scale-Sum of Boxes (CDR-SB) scores, though these findings are preliminary and durable disease-modifying effects remain to be established (54).

### 3.2 Transcranial alternating current stimulation

Transcranial alternating current stimulation (tACS) delivers low-intensity alternating current through scalp electrodes, with neural oscillation entrainment as its core mechanism: exogenous current at a specific frequency drives synchronized neuronal firing, thereby correcting pathological oscillatory disturbances (60–62). In AD patients, 40-Hz tACS targets key regions (precuneus, temporal lobe, hippocampus) to exogenously induce and enhance gamma-band (20–50 Hz) oscillations, reversing the characteristic gamma reduction in AD (63). The efficacy of oscillation entrainment—and consequently glymphatic modulation—is directly determined by electric field distribution, stimulation target, and treatment duration (64, 65).

tACS might modulate the glymphatic system through multiple indirect pathways. In healthy individuals and animal models, several tACS researches reported that it increased regional cerebral perfusion, altered functional connectivity, enhanced theta–gamma coupling, and improved cholinergic measures. These outcomes provided potential neurovascular and neurophysiological mechanisms through which tACS could influence CSF dynamics, though these are indirect physiological proxies rather than direct evidence of glymphatic enhancement or clinical benefit in AD (66–68).

A remote-supervised, home-based 40-Hz tACS study in AD patients found significant improvements in memory index scores following the acute intervention, with effects maintained during a 2–3 month washout period. Among three patients who completed EEG recordings, the theta/gamma ratio in the left angular gyrus was significantly reduced, which may be related to the correction of abnormal rhythms and provide rhythmic driving support of CSF-ISF exchange (69). In healthy adults, tACS targeting the right inferior frontal gyrus improved inhibitory control and cognitive flexibility while enhancing functional connectivity between this region and the frontopolar area (70). Similar to rTMS, *APOE* genotype and baseline cognitive impairment level are important predictors of 40-Hz tACS efficacy, with *APOE*ϵ4 carriers showing specific response characteristics that have the potential to guide individualized intervention strategies, though these findings remain exploratory and require confirmation in larger, adequately powered trials (64). These findings remain preliminary and require validation in independent, adequately powered studies before any clinical application.

### 3.3 Visual 40-Hz light stimulation

40-Hz light flicker therapy induces synchronized gamma oscillations in brain neurons via retinal stimulation, which in turn coordinates neuronal, glial, and cerebrovascular activity (**Figure 4**). Given the close association between gamma oscillations and glymphatic function, this technique provides a rhythmic basis for glymphatic modulation (71, 72). 40-Hz light flicker enhances glymphatic influx (CSF entry into the brain parenchyma via periarterial spaces and astrocytic AQP4 channels) and efflux (clearance of ISF and metabolic wastes from the parenchyma via perivenous drainage routes) through multidimensional synergistic effects.

**Figure 4:**
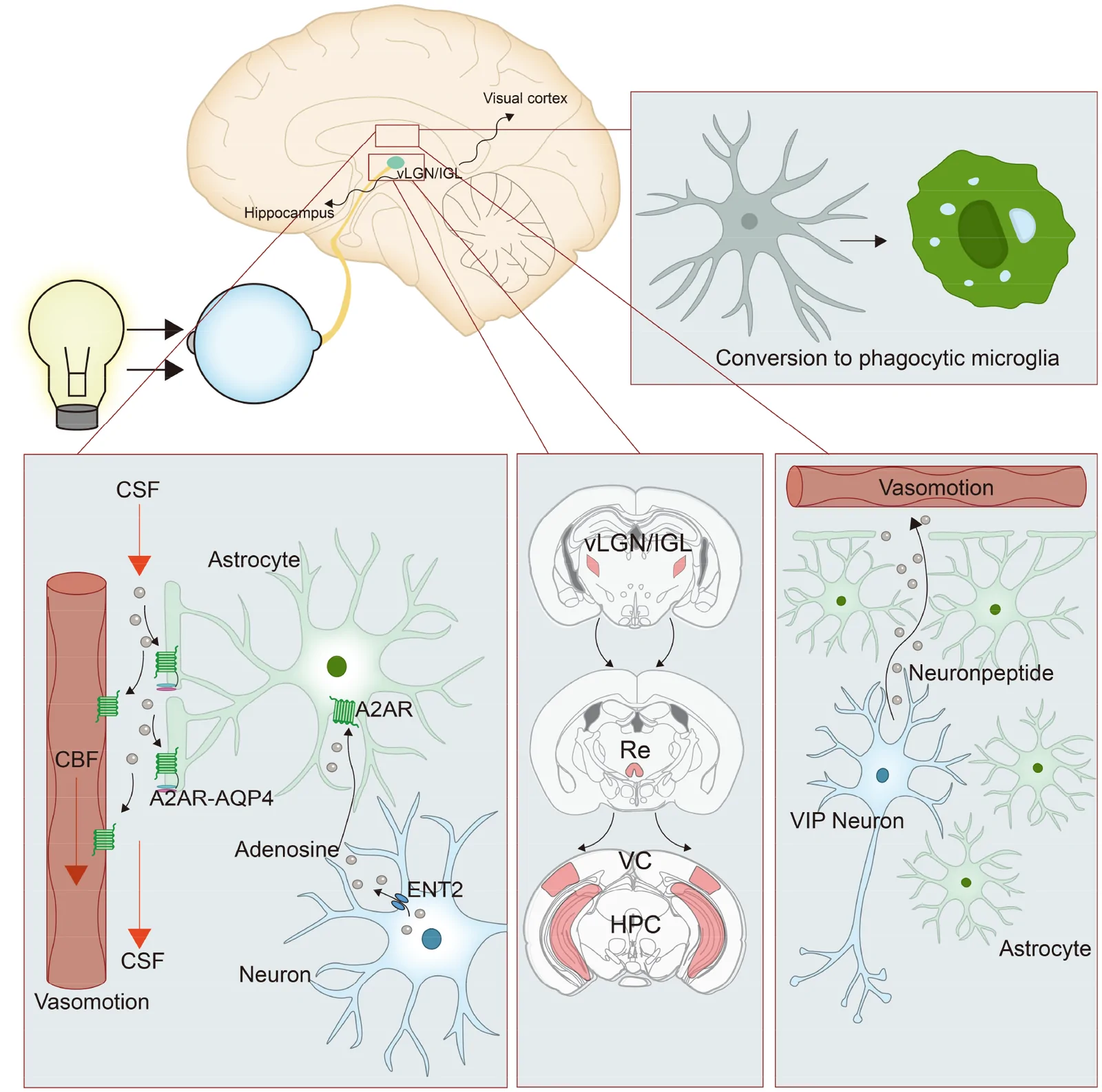
Mechanisms of 40-Hz light stimulation in glymphatic modulation. 40-Hz light stimulation exerts multi-modal effects to enhance glymphatic clearance. Visual input is relayed via the visual pathway, including the ventral lateral geniculate nucleus/intergeniculate leaflet(vLGN/IGL), to multiple brain regions. Key mechanisms include: (1) activation of the adenosine-A2A receptor(A2AR)-AQP4 axis, where increased CSF adenosine binds astrocytic A2AR to promote polarized AQP4 distribution and vasomotion, driving CSF-ISF exchange; (2) entrainment of gamma oscillations across the nucleus reuniens(Re), visual cortex(VC), and hippocampus(HPC); (3) modulation of vasomotion and neuropeptide release from VIP-expressing interneurons, enhancing vascular dynamics; and (4) promotion of microglial conversion to a phagocytic phenotype, which reduces pathological protein burden and alleviates glymphatic clearance load.

A2A receptor (A2AR) is a G protein-coupled receptor that binds adenosine and is highly expressed in brain tissues including the striatum and hippocampus (73). Equilibrative nucleoside transporter 2 (ENT2) primarily mediates nucleoside uptake at the astrocytic membrane, thereby modulating extracellular adenosine concentrations at the cell surface. It is essential for nucleotide synthesis via the salvage pathway in cells such as neurons, which lack the *de novo* biosynthetic pathway for nucleotides. By modulating extracellular adenosine concentrations, nucleoside transport also plays a pivotal role in the regulation of numerous physiological processes, including vasodilation and blood flow regulation (74). In murine studies, a central mechanism involves the adenosine–A2AR signaling axis. 40-Hz light stimulation significantly elevates CSF adenosine levels by balancing ENT2-mediated adenosine efflux at the astrocytic membrane. Adenosine then binds to A2AR on astrocytic surfaces, promoting polarized AQP4 distribution along vascular endfeet, thereby strengthening glymphatic exchange. Blockade of either ENT2 or A2AR completely abolishes this effect, confirming this axis as the core pathway for light-induced glymphatic modulation, with adenosine’s vasodilatory effects potentially representing an additional parallel mechanism (73–75). These mechanistic observations, however, are derived from animal studies and require clinical validation to confirm their relevance to human therapy.

Additional pathways have also been identified. In 5xFAD mice, 40-Hz blue light activates the ventral lateral geniculate nucleus/intergeniculate leaflet-nucleus reuniens (vLGN/IGL-Re) visual circuit, reverses hippocampal AQP4 depolarization, facilitates CSF influx along PVS, and accelerates Aβ clearance from ISF (76). It also increases AQP4 polarization and acts synergistically with meningeal lymphatic dilation to improve drainage efficiency. Additionally, preclinical evidence suggests that 40-Hz light activates vasoactive intestinal peptide (VIP) interneurons and regulates arterial pulsation amplitude to promote CSF influx in animal models.

Moreover, in AD mouse models, 40-Hz light drives microglia toward a phagocytic phenotype, enhances their co-localization with Aβ plaques, strengthens Aβ phagocytosis, and may alleviate the waste-clearance burden on the glymphatic system. Activated microglia secrete anti-inflammatory factors that improve the neuroinflammatory microenvironment and indirectly promote glymphatic recovery in these preclinical studies (71, 77). Combination with exercise further reduces pro-inflammatory cytokine levels and inhibits excessive glial activation (78–80).

In 5xFAD and APP/PS1 mice, 40-Hz light flicker significantly improves CSF influx into the brain parenchyma via periarterial spaces and ISF efflux from the parenchyma via perivenous drainage routes, as reflected by expanded tracer distribution and accelerated clearance, restores normal AQP4 polarity, and dilates meningeal lymphatic vessels (76). It reduces cerebral Aβ40 and Aβ42 levels and alleviates plaque burden in aged AD mice, while also ameliorating motivational deficits and spatial learning and memory impairments—benefits closely associated with enhanced glymphatic function and Aβ clearance (71, 76).

Plasma neurodegenerative biomarkers including Aβ40, Aβ42, total tau, and glial fibrillary acidic protein show disease-specific associations with glymphatic and meningeal lymphatic functional indices (81). A *post-hoc* exploratory analysis within a phase III clinical trial (NCT03543878) using CSF proteomic analysis found that 110 relevant proteins were significantly differentially expressed in prodromal AD individuals after 8 weeks of daily 1 hour treatment, indicating modulation of multiple pathways associated with the cellular phenotypes of AD, though these proteomic changes do not by themselves demonstrate clinical efficacy or safety.

### 3.4 Transcranial photobiomodulation

Transcranial photobiomodulation (tPBM) uses near-infrared (NIR) light within the 600–1300 nm range to target brain neurons, glial cells, and mitochondria. Its core principle relies on photochemical effects on mitochondrial cytochrome c oxidase (CCO), promoting ATP production and reducing ROS accumulation, thereby modulating neuronal metabolism and inflammatory responses (82, 83).

Regarding glymphatic modulation, preclinical and healthy human studies have reported direct effects have been observed. tPBM can directly alter intracranial CSF flow dynamics—a 4-minute intervention increases intracranial CSF volume by 3% to more than 20% in healthy adults, regulating CSF-ISF exchange efficiency (84). Optical coherence tomography reveals that tPBM significantly reduces cerebral Aβ plaque deposition in animal models and may point to potential enhancement of glymphatic function (85). Indirect effects are equally important. tPBM decreases hippocampal pro-inflammatory cytokine levels in aged rats, promotes microglial conversion to an anti-inflammatory phenotype, prevents inflammation-mediated perivascular space stenosis, and maintains glymphatic structural integrity (82, 86, 87). It also alters EEG power, enhances γ/β wave power in healthy brains, and optimizes frontoparietal functional network connectivity (88). At the microscopic level, tPBM induces tubulin conformation transition from α-helix to β-sheet, reducing microtubule stability and enhancing dynamics to facilitate microtubule remodeling and turnover (89). At the genetic level, it downregulates expression of AD risk genes including BACE1 and PSEN2, an effect associated with reduced Aβ load in preclinical models, though these findings are derived from animal and healthy subject studies and do not by themselves demonstrate clinical efficacy in AD (82).

Clinically, tPBM increases cerebral activity in irradiated areas of MCI patients, with significant improvements in visual working memory, MoCA scores, and depressive symptoms (90, 91). In healthy older adults, tPBM improves 3-back working memory accuracy and response time, enhances global and local efficiency of frontoparietal functional networks, with accuracy improvement positively correlated with left prefrontal functional connectivity (92, 93). A single-case report of a 59-year-old APOEϵ4-positive mild AD patient with insulin-dependent type 2 diabetes showed significantly improved metabolic indicators and normalized MoCA scores after 10 weeks of ketogenic diet combined with tPBM, correlating with possible genetic specificity of tPBM efficacy.

### 3.5 Focused ultrasound

Low-intensity focused ultrasound (LIFU) can directly drive CSF influx into PVS and improve transport efficiency of both small and large molecules, as reflected by accelerated tracer clearance and greater tracer dispersion within the brain parenchyma, thereby providing a physical driving force for waste clearance (94, 95). The most prominent mechanism involves LIFU combined with microbubbles (FUS/MB) (96). This combination enables transient blood-brain barrier (BBB) opening, facilitating both entry of peripheral therapeutic agents (e.g., anti-Aβ antibodies) into brain parenchyma and potential excretion of brain wastes into the bloodstream, thereby relieving glymphatic clearance burden (97, 98).

In AD model mice, low-intensity pulsed ultrasound activates microglia in AD model mice and enhances their phagocytic capacity toward Aβ plaques. Activated microglia secrete anti-inflammatory cytokines to improve the PVS inflammatory microenvironment which is linked to glymphatic structural integrity in these animal studies (99). LIFU also remodels brain functional connectivity in AD patients, restores gamma oscillations, improves neural circuit and cerebral hemodynamic synchrony, and coordinates glymphatic drainage Finally, LIFU upregulates endothelial nitric oxide synthase (eNOS) expression in AD mouse brains, promotes vascular endothelial repair and vasodilation, increases cerebral blood flow (CBF), and strengthens the driving force for glymphatic drainage (99).

Large animal studies support clinical translation. An acoustic-transparent cranial implant in a sheep model enables precise FUS-mediated BBB opening and defines a safe and effective pressure range, providing parameter optimization references for glymphatic-targeted AD interventions (100). In an early feasibility study, MR-guided FUS targeting the hippocampus and entorhinal cortex in early AD patients achieves safe, transient, and repeatable BBB opening. Expanding the treatment field to the frontoparietal lobe resulted in a 14% reduction in Aβ plaque PET signal, an imaging biomarker finding rather than a direct measure of cognitive or clinical benefit, with no additional cognitive decline and no serious adverse events during 6–12 months of follow-up (101). Notably, preclinical evidence suggests that LIFU may increase intracranial hemorrhage risk in the setting of acute cerebrovascular injury, although this finding requires further validation and may not directly translate to clinical contexts (102). Overall, FUS-mediated BBB opening holds promising translational potential, though clinical efficacy, durability, and cognitive benefits remain to be established in larger, adequately powered trials with clinical endpoints.

### 3.6 Regular aerobic exercise

Persistent moderate-to-vigorous physical activities (running, swimming, interval training) trigger multidimensional physiological effects that modulate the glymphatic system. Exercise promotes astrocytic conversion toward an A2 neuroprotective phenotype characterized by more pronounced AQP4 polarization, and upregulates transcription of AQP4 anchoring proteins (Lama1, Dp71), thereby reducing AQP4 depolarization (103, 104). It is well established that exercise enhances arterial pulsation, which directly drives CSF influx into brain parenchyma along PVS (105). Long-term aerobic exercise also optimizes meningeal lymphatic drainage function, increasing vessel diameter and improving drainage capacity in both AD mouse models and humans in AD mouse models and general/healthy human cohorts (106, 107). These effects collectively accelerate CSF-ISF exchange and enhance glymphatic clearance efficiency in these populations.

Aerobic exercise attenuates excessive astrocytic and microglial activation in aged mice and APP/PS1 models, reducing pro-inflammatory cytokine release (105, 108). One study revealed that in 5×FAD mice, thrombospondin-1 (TSP-1) secreted by reactive astrocytes after one month of treadmill exercise inhibited meningeal lymphangiogenesis by binding to CD36 and CD47. However, long-term treadmill exercise downregulates EAF2 expression in astrocytes, suppressing p53-mediated TSP-1 transcription and relieving meningeal lymphatic inhibition via the EAF2-p53-TSP-1 axis (107). Chronic inflammation or aging can activate astrocytic TRPV4 channels, leading to excessive calcium accumulation and disrupted AQP4 polarization. Exercise inhibits TRPV4 expression and calcium signaling overactivation, indirectly promoting AQP4 polarization and glymphatic clearance. It must be pointed out that the animal model used in this study is aged mice with colitis, therefore the effects on glymphatic function are of limited direct relevance to AD (109). Notably, while these mechanistic and physiological observations are encouraging, AD-specific clinical outcomes, including cognitive benefit or disease modification, remain to be demonstrated.

### 3.7 Sleep optimization and respiratory regulation

Given the inherent circadian rhythm of the glymphatic system, improving sleep architecture—particularly NREM sleep and slow-wave sleep (SWS)—holds great therapeutic potential for AD. Sleep optimization leverages characteristic changes in brain physiological states across sleep cycles to modulate glymphatic function (110). In patients with obstructive sleep apnea (OSA), interventions such as continuous positive airway pressure (CPAP) correct chronic intermittent hypoxia and sleep fragmentation, restoring normal sleep architecture (111). During NREM sleep, norepinephrine oscillations mediate cerebrovascular vasomotion, which synchronizes with CSF flow and promotes extracranial drainage via meningeal lymphatic vessels (27). OSA-induced chronic hypoxia disrupts norepinephrine homeostasis, leading to reduced DTI-ALPS scores and impaired visual memory (112, 113). By reversing hypoxia, CPAP restores this regulatory axis and improves DTI-ALPS scores in OSA patients (27, 114, 115). Furthermore, CPAP increases intracranial pressure (ICP) pulse amplitude, directly accelerating CSF flow velocity and enhancing regional glymphatic transport as reported in OSA and healthy populations (116). CPAP therapy also stabilizes PVS volume in the basal ganglia of OSA patients, preventing its progression to pathological dilatation (111). Similar to other interventions, sleep optimization and respiratory regulation attenuate excessive glial activation, suppress neuroinflammation, and synergistically promote metabolic waste clearance (117–119). Notably, while these physiological observations in OSA patients and animal models are encouraging, the relevance of CPAP-induced glymphatic changes to AD pathology and clinical outcomes in AD populations remains to be established.

## 4 Glymphatic biomarkers

The glymphatic system exerts intricate functions, and no single examination modality is capable of fully depicting all facets of glymphatic transport. Each existing detection method possesses distinct advantages and limitations.

Intrathecal contrast-enhanced MRI most directly maps the distribution and clearance patterns of CSF tracers in the human brain. Nevertheless, this modality requires lumbar puncture, off-label administration of gadolinium-based contrast agents, and lengthy scanning sessions (120). DTI-ALPS index is non-invasive, clinically accessible and repeatable. However, it serves only as an indirect, region-specific metric, whose measurements are influenced by white--matter architecture and imaging parameter configurations (121). Dynamic contrast-enhanced imaging yields spatially resolved tracer kinetic data, but it is time-consuming and route-dependent. Intravenous contrast signals predominantly reflect BBB transport rather than intrinsic glymphatic fluid flow. Diffusion and free-water methods are contrast-free approaches that quantitatively measure interstitial water mobility, but they fail to differentiate impaired fluid clearance from water disturbances induced by cerebral edema, inflammation or brain tissue atrophy (122). PVS burden is easily measured on routine structural MRI, yet enlarged spaces are nonspecific and indicate morphology rather than transport (123). CSF-BOLD coupling non-invasively captures sleep-related coordination between neural, vascular, and CSF oscillations, but depends strongly on arousal, vascular physiology, and analysis methods (124). Phase-contrast MRI enables quantitative assessment of bulk directional CSF flow velocity and pulsatility, though its spatial resolution is insufficient for microscopic PVS exchange (120). In summary, existing imaging approaches for assessing glymphatic function differ substantially in invasiveness, spatial resolution, measured parameters, and clinical applicability. A multimodal imaging strategy is therefore needed to comprehensively and accurately evaluate glymphatic system function in humans.

## 5 Conclusions and perspectives

In light of current evidence, the glymphatic system perhaps can be a potential adjunct bridging the molecular pathology of Alzheimer’s disease and the physiological dynamics of cerebral waste clearance. Unlike traditional therapeutic strategies that focus on single pathogenic targets such as Aβ or tau, non-invasive modulation of the glymphatic system, though its therapeutic efficacy, optimal dosing, durability, and clinical relevance remain uncertain, offers a different perspective: rather than directly attacking pathological species, it seeks to restore the brain’s intrinsic clearance machinery. This shift from a “target-and-eliminate” approach to a “restore-and-maintain” strategy may offer potential benefits given the multifactorial nature of AD, where impaired clearance often precedes and perpetuates pathological accumulation. The seven intervention modalities reviewed here—rTMS, tACS, 40-Hz light stimulation, tPBM, LIFU, aerobic exercise, and sleep optimization—each engage distinct yet overlapping nodes within the glymphatic network, including arterial pulsation, CSF-turnover, AQP4 polarization, meningeal lymphatic drainage, and circadian regulation. Collectively, they demonstrate that the glymphatic system is not a passive conduit but a dynamically regulatable biological system amenable to diverse physical, electromagnetic, optical, and behavioral inputs.

Although these interventions are categorized as non-invasive, they are not entirely devoid of therapeutic risks. Such risks encompass seizure episodes, severe headache and elevated depression risk associated with rTMS; skin burns induced by tACS; photosensitive epilepsy or migraine triggered by light stimulation; low-temperature thermal injuries arising from tPBM; intracranial hemorrhage, cerebral edema, BBB disruption, alongside microbubble injection-related hypersensitivity and vasculitis linked to FUS; physical limitations pertaining to exercise interventions; as well as intolerance to CPAP and catheter-associated pneumonia. Encouragingly, the preliminary safety profiles of these non-invasive neuromodulation strategies are consistent with our expectations. Aside from infrequent mild adverse sensations including transient stinging, scalp irritation, slight visual disturbances and mild headache, no severe treatment-related adverse events have been documented. One study described a single case of transient hippocampal edema following FUS administration, which resolved spontaneously within 72 hours without any subsequent behavioral abnormalities. Future studies ought to continuously monitor the occurrence of such serious adverse events to guarantee the overall safety.

A unique insight emerging from this synthesis is that the effectiveness of non-invasive glymphatic modulation may depend less on the specific technology employed than on its ability to engage multiple mechanistic pathways concurrently. Single-target approaches targeting a single driver—such as enhancing arterial pulsation alone or upregulating AQP4 expression in isolation—may yield suboptimal effects, whereas interventions that simultaneously influence intrinsic drivers (norepinephrine oscillations, AQP4 polarity) and extrinsic modulators (meningeal lymphatic function, intracranial pressure gradients) appear to produce more robust outcomes. This suggests hypotheses that future research should prioritize combinatorial or multimodal strategies, such as pairing sleep optimization with 40-Hz light stimulation or combining aerobic exercise with rTMS, to achieve synergistic glymphatic enhancement. Beyond these promising hypotheses, combinatorial therapeutic regimens carry the potential to escalate treatment burden or raise the risk of adverse reactions, and do not necessarily translate into superior clinical efficacy. Nevertheless, given their promising clinical prospects, further investigations are warranted to fill the current research gaps, including factorial trial design, dose-finding studies, interaction analyses, safety monitoring, glymphatic function assessment, as well as evaluation of additive versus synergistic effects. Furthermore, the observation that APOEϵ4 carriers, despite exhibiting poorer baseline glymphatic function, show greater responsiveness to certain non-invasive interventions—notably rTMS and tACS—in preliminary studies, raising the intriguing possibility of genotype-guided personalized modulation. This inverse relationship warrants further investigation and requires independent replication in larger, prespecified trials before it can inform patient stratification. This inverse relationship—greater therapeutic reserve in genetically vulnerable populations—warrants further investigation and could redefine patient stratification in future clinical trials.

Looking forward, several priorities should guide the next generation of research. First, the field must move beyond reliance on indirect indicators such as DTI-ALPS or cognitive scores and develop direct, real-time, and quantitative measures of human glymphatic activity. Emerging techniques including intrathecal contrast-enhanced MRI, ultrafast ultrasound imaging of PVS flow, and non-invasive near-infrared spectroscopy tracking of CSF dynamics hold promise but require validation and standardization. Second, mechanistic studies at the molecular level are urgently needed. While we know that AQP4 polarization is critical, the precise signaling pathways—from neurotransmitter receptors to cytoskeletal rearrangements—through which non-invasive stimuli regulate AQP4 trafficking remain largely unexplored. Similarly, the differential effects of various frequencies, intensities, and durations of stimulation on meningeal lymphatic contractility and valve function represent a rich area for investigation. Third, clinical trial design must evolve from proof-of-concept to precision medicine. Screening strategies based on glymphatic-related biomarkers (e.g., baseline AQP4 expression, *APOE* genotype, or CSF adenosine levels) could reduce heterogeneity and increase statistical power. Long-term, sequential, and home-based intervention protocols should be developed to assess durability of effects and real-world feasibility. Finally, interdisciplinary collaboration among neuroengineers, molecular neuroscientists, radiologists, and neurologists will be essential to translate mechanistic insights into clinically deployable devices and protocols.

In conclusion, non-invasive modulation of the glymphatic system represents a promising frontier in AD therapeutics, one that aligns with the emerging recognition of Alzheimer’s as a disorder of impaired waste clearance as much as one of protein aggregation. By harnessing the brain’s own clearance infrastructure through safe, repeatable, and accessible interventions, this perspective offers not only a complement to existing immunotherapies but potentially a preventative target applicable in prodromal or even preclinical stages, holding translational potential for the prevention, treatment or adjuvant therapy of AD.

## 6 Search strategy and selection criteria

We searched the PubMed database using keywords and their combinations including, but not limited to: Alzheimer’s disease, AD, glymphatic, AQP4, PVS, CSF-ISF exchange, repetitive transcranial magnetic stimulation, transcranial alternating current stimulation, 40-Hz light, photobiomodulation, focused ultrasound, aerobic exercise, and sleep optimization. Retrieved citations were imported into EndNote 21 for initial screening based on titles and abstracts, and the final inclusion of references was determined by full-text evaluation.
